# Swing-Out of the β3 Hybrid Domain Is Required for αIIbβ3 Priming and Normal Cytoskeletal Reorganization, but Not Adhesion to Immobilized Fibrinogen

**DOI:** 10.1371/journal.pone.0081609

**Published:** 2013-12-09

**Authors:** Ming Cheng, Jihong Li, Ana Negri, Barry S. Coller

**Affiliations:** 1 Allen and Frances Adler Laboratory of Blood and Vascular Biology, The Rockefeller University, New York, New York, United States of America; 2 Department of Structural and Chemical Biology, Icahn School of Medicine at Mount Sinai, New York, New York, United States of America; Thomas Jefferson University, United States of America

## Abstract

Structural and functional analyses of integrin αIIbβ3 has implicated swing-out motion of the β3 hybrid domain in αIIbβ3 activation and ligand binding. Using data from targeted molecular dynamics (TMD) simulations, we engineered two disulfide-bonded mutant receptors designed to limit swing-out (XS-O). XS-O mutants cannot bind the high Mr ligand fibrinogen in the presence of an activating mAb or after introducing mutations into the αIIb subunit designed to simulate inside-out signaling. They also have reduced capacity to be “primed” to bind fibrinogen by pretreatment with eptifibatide. They can, however, bind the small RGD venom protein kistrin. Despite their inability to bind soluble fibrinogen, the XS-O mutants can support adhesion to immobilized fibrinogen, although such adhesion does not initiate outside-in signaling leading to normal cytoskeletal reorganization. Collectively, our data further define the biologic role of β3 hybrid domain swing-out in both soluble and immobilized high Mr ligand binding, as well as in priming and outside-in signaling. We also infer that swing-out is likely to be a downstream effect of receptor extension.

## Introduction

Integrins belong to a cell adhesion molecular family that mediates cell-cell and cell-extracellular matrix interactions [1]. They signal bidirectionally through long-range allosteric changes, with proteins binding to the cytoplasmic domains initiating inside-out signaling and ligands binding to the extracellular domain initiating outside-in signaling [2].

Integrin αIIbβ3 is expressed on megakaryocytes and platelets and on cells early in hematopoietic stem cell development [3]. Platelet αIIbβ3 contributes to hemostasis by supporting platelet aggregation at sites of vascular injury and pathological thrombosis by supporting platelet aggregation in atherosclerotic arteries, with the latter leading to myocardial infarction and stroke [4], [5]. Physiological agonists such as ADP or thrombin initiate inside-out platelet signaling and induce αIIbβ3 conformational changes that result in the binding of multimeric ligands, such as fibrinogen and von Willebrand factor. The simultaneous binding of either of these ligands to αIIbβ3 receptors on two different platelets then results in platelet aggregation via crosslinking of platelets. Ligand binding also initiates outside-in signaling, leading to cytoskeletal reorganization and enhanced secretion [6]. The lifelong bleeding disorder Glanzmann thrombasthenia is an autosomal recessive disease in which patients either lack or have abnormal αIIbβ3 receptors [3].

Similar to other integrins, activation of, and ligand binding to αIIbβ3 is associated with large-scale global conformational rearrangements [2], [7]–[13]. Extensive structural and functional data have shown that αIIbβ3 exists in at least three different conformations: a bent conformation with a closed headpiece (i.e., the β3 hybrid domain abuts the αIIb β-propeller), an extended conformation with a closed headpiece, and an extended conformation with an open headpiece (i.e., the β3 hybrid domain swings out from the αIIb β-propeller by 60–70°). Although all three conformations are capable of binding small ligands, the bent, closed conformation has low affinity for macromolecular physiologic ligands whereas both the extended, closed and extended, open conformations are associated with higher affinity for these ligands. The transition from the bent to the extended conformation, and from the closed to open conformation, can be achieved by adding peptides that contain the cell recognition Arg-Gly-Asp (RGD) sequence, which bind to the ligand binding site at the junction between the two head domains [8], [13]. These peptides are thought to induce the open conformation by altering the structure around the β3 metal binding sites, leading to the downward movement of the α7 helix of the β I domain (β3 Inserted domain) (which connects the β I domain to the hybrid domain), which, in turn, initiates the swing-out motion of the hybrid domain away from αIIb [8]. Initial experimental support for the swing-out conformation having high ligand affinity came from data demonstrating that stabilizing the open headpiece conformation by introducing a disulfide bond in the β I domain [14] or engineering a new N-glycosylation site into the hybrid-β I domain interface to wedge the hybrid domain away from the β I domain [15] creates constitutively active receptors that do not require inside-out signaling to induce ligand binding.

To define better the relative contributions of αIIbβ3 extension and β3 hybrid domain swing-out to high affinity ligand binding, several investigators have engineering disulfide bonds into the receptor to limit or stabilize specific motions (Table 1). These cross-links were designed to limit: both extension and swing-out (αIIbR320C/β3R563C) [9], swing-out (β3T329C/A347C [14] and αIIbD319C/β3V359C [17]), αIIb extension (R597CY645C) [16], or β3 extension (S367C/S551C, G382C/T564C, and V332C/S674C) [17]. Other more recent studies have introduced mutations to induce or facilitate β3 swing-out by: inducing β3 extension by shortening a key loop in the β3 I-EGF1 domain [18]; both removing the β I-β T (β3 Tail domain) interface and creating two new N-linked glycosylation sites (V332N/S674N/K676T) [17]; or inducing αIIβ extension by creating N-linked glycosylation sites in the αIIb thigh domain near the genu (Q595N/R597T; D589N/H591T) [17].

**Table 1:** Cysteine mutations in αIIbβ3 designed to limit or stabilize conformational changes.

Cysteine Amino Acid Mutations	Affected Domains	Functional Consequences	Rescued by Activating Mutations	References
αIIbR597C/Y645C	αIIb thigh and calf domain	Unable bind to soluble Fbg (fibrinogen), but can adhere to immobilized Fbg	β3N339S rescues	[16]
β3S367C/S551C	β3 hybrid/EGF-3	Unable to bind soluble Fbg		[17]
β3G382C/T564C	β3 hybrid/EGF-4	Unable to bind soluble Fbg		[17]
β3V332C/S674C	β3 βI/β3 Tail	Unable to bind soluble Fbg		[17]
αIIbD319C/V359C	αIIb β-propeller/β3 hybrid	Unable to bind soluble Fbg, but can bind mAb OP-G2	αIIb Q595N/R597T does not rescue	[17]
αIIbR320C/β3 R563C	αIIb β-propeller/β3 EGF-4	Unable to bind soluble Fbg		[9]
β3T329C/A347C	β3 βI	Unable to bind Fbg and adhere to immobilized Fbg		[14]
β3V332C/M335C	β3 βI	Constitutively binds Fbg and adheres to immobilized Fbg		[14]
αIIbK321C/β3 E358C and αIIbK321C/β3 R360C	αIIb β-propeller/β3 hybrid	Unable to bind soluble Fbg, but can bind snake venom protein kistrin and adhere to immobilized Fbg	αIIb F992A/F993A does not rescue	This paper

These cross-links were designed to limit: both extension and swing-out (αIIbR320C/β3R563C) [9], swing-out (β3T329C/A347C [14] and αIIb319/β3V359C [17]), αIIb extension (R597C-Y645C) [16], or β3 extension (S367C/S551C, G382C/T564C, and V332C/S674C) [17]. A β3 V332C/M335C disulfide mutant was designed to induce swing-out.

In a previous study we employed targeted molecular dynamics (TMD) simulations to study the pathway of the swing-out transition from the unliganded, closed to the liganded, open conformation of β3 integrins [19]. Stereochemically feasible pathways with candidate intra-domain and inter-domain interactions responsible for β3 integrin activation were explored and specific contacts were identified that are broken early during the swing-out process. Thus, creation of a new disulfide bond between these residues would be expected to prevent the normal swing-out mechanism. In this study we assessed the effect of creating two different mutant receptors, each of which contained two new cysteine residues designed to create a new disulfide bond that would prevent swing-out. Our data support and extend those of Kamata et al. [17] who studied a similar mutant receptor (αIIbβ D319C/V359C). We also observed inhibition of the binding of large, but not small, activation-dependent soluble ligands. In addition, we were unable to rescue the abnormality in soluble ligand binding by introducing mutations into the αIIb subunit designed to simulate inside-out signaling. Moreover, we found that preventing swing-out prevented ligand binding induced by “priming” the receptor with low molecular weight ligands, but did not affect the receptor's ability to support adhesion to immobilized fibrinogen. Finally, we found that the mutations inhibited outside-in signaling after cell adhesion.

## Materials and Methods

### Reagents and antibodies

The αIIbβ3 activating mAb PT25-2 [20] was generously provided by Dr. Makota Handa (Keio University, Tokyo, Japan), the anti-LIBS (Ligand Induced Binding Site) mAb AP5 [21] was generously provided by Dr. Peter Newman (Blood Center of Wisconsin), and the anti-αVβ3 mAb LM609 [22] was generously provided by Dr. David Cheresh (University of California at San Diego). The mAbs 10E5 (anti-αIIbβ3) [23], 7E3 (anti-αIIbβ3 + αVβ3) [24], PMI-1 (anti-αIIb), and 7H2 (anti-β3) [25] were produced at the National Cell Culture Center. FITC-PAC-1 (anti-activated αIIbβ3) [26] was purchased from BD Biosciences (San Jose, CA). Anti-vinculin murine mAb clone 7F9 was from Millipore. The disintegrin kistrin (rhodostomin) from the venom of Agkistrodon rhodostoma [27] was the gift of Dr. Tur-Fu Huang (Taiwan University). Alexa488 labeling of kistrin, 10E5, and AP5 was carried out according to the manufacturer's instructions (Invitrogen). Alexa488-fibrinogen was obtained from Invitrogen. Human thrombin was obtained from Enzyme Research Laboratories.

### Targeted molecular dynamics (TMD) simulations

TMD simulations were performed to simulate the swing-out motion of the β3 hybrid (and PSI) domains as previously described [19]. The head and upper leg regions of αIIbβ3 were simulated, including the β-propeller and thigh domains of αIIb and the β I, hybrid, and PSI domains of β3. Amino acid numbering is based on the mature protein without the leader sequence.

### Site-directed mutagenesis

Based on the results of the TMD simulations, mutants designed to prevent swing-out (XS-O), αIIbK321C-β3E358C (321–358) and αIIbK321C-β3R360C (321–360) were generated using the QuikChange XL Site-directed Mutagenesis Kit (Stratagene, La Jolla, CA) according to the manufacturer's instructions. The αIIbF992A/F993A-β3 double mutant receptor (αIIbFFβ3) and the combined XS-O mutants FF321/358 (αIIbK321C/F992A/F993A-β3E358C) and FF321/360 (αIIbK321C/F992A/F993A-β3R360C) were also prepared. The mutant cDNAs were all sequenced to confirm that the mutations were introduced as predicted.

### Cell transfection and generation of stable cell line

Human embryonic kidney (HEK) 293 cells were transfected with either normal or mutant cDNA using Calphos mammalian transfection reagents (Clontech). Cells were selected in 80 µg/ml G418 followed by sorting based on their binding of Alexa488-conjugated mAb 10E5, which binds to the cap region of the αIIb β-propeller domain [8]. Although HEK293 cells make variable amounts of αV, which can combine with transfected β3 to form αVβ3, we found little or no αV on cells expressing normal αIIbβ3 (Fig. S1).

### Assessment of disulfide bond formation from XS-O mutants by mass spectrometry

Cells expressing XS-O mutants and normal αIIbβ3 were lysed using 1% Triton X-100, followed by immunoprecipitation with mAb 10E5. After resolving the proteins on a non-reduced SDS-PAGE gel, the purified protein bands from XS-O mutant 321/358 corresponding to the disulfide-bonded αIIbβ3 heterodimer and the individual αIIb or β3 subunits were cut out and digested overnight with trypsin (2 µg/ml; Promega Madison, WI). Comparable purified protein bands from XS-O mutant 321/360 were digested with trypsin overnight, followed by digestion with endoproteinase Asp-N (2 µg/ml; Roche; Basel, Switzerland) for another two days. Samples were analyzed by LC-MS/MS using a nano-reversed phase column coupled online with an LTQ-Orbitrap mass spectrometer (ThermoFisher; Waltham, MA).

### Spreading of adherent cells expressing normal αIIbβ3 and XS-O mutants

Chamber slides were coated with 20 µg/ml fibrinogen for 1 hr and then washed and blocked with HBMT. Cells (1.5×10^5^ cells/ml, 200 µl) in HBMT buffer with 2 mM Ca^2+^ and 1 mM Mg^2+^ were added to each chamber. In some experiments, 1 mM Mn^2+^ was used to activate the cells. Cells were allowed to adhere to fibrinogen for 2 hr at 37°C. After washing with PBS, adherent cells were fixed with 4% formaldehyde in PBS and permeabilized with 0.5% Triton X-100 in PBS. After washing, adherent cells were stained with Alexa488-7H2 for 20 min, washed, and stained for F-actin with rhodamine phalloidin (Cytoskeleton Inc. Denver, USA) for 30 min. The cells were then washed and dried at room temperature (RT). A drop of anti-fade mounting medium (Dako, Carpinteria, CA) was added to the center of the chamber, a cover slip was placed on top of the cells, and the cover slip was sealed with nail polish. The specimens were imaged using a wide-field fluorescence/brightfield/DIC microscope (Zeiss) with a 40X objective. Image J (NIH) was used to analyze cell spreading.

For double staining of vinculin and actin, cells were allowed to adhere for 1 hr to wells pre-coated with 20 µg/ml fibrinogen at RT. After fixation and permeabilization, cells were reacted with an anti-vinculin antibody, followed by staining with a secondary FITC-anti-mouse antibody and TRITC-conjugated phalloidin simultaneously according to the manufacturer's protocol (Millipore). The specimens were imaged using a DeltaVision Image Restoration Microscope (Olympus) with a 60X objective. For time-lapse microscopy, live cells were added to cover-glass chamber slides pre-coated with 20 µg/ml fibrinogen at RT and differential interference contrast (DIC) images were obtained every 2 min for 1 hr using the DeltaVision microscope with a 20X objective.

### Priming

Harvested cells in HBMT containing 1 mM MgCl_2_ and 2 mM CaCl_2_ (10^5^ cells/ml, 100 µl) were incubated with buffer, EDTA (10 mM), eptifibatide (1 µM), the peptide RGDS (100 µM), RUC-1[28], [29], or RUC-2[30] for 20 min at RT; fixed with 2% paraformaldehyde for 40 min at RT; incubated with 800 µl 5 mM glycine for 5 min; washed X 4 and resuspended in HBMT buffer containing 1 mM MgCl_2_ and 2 mM CaCl_2_; incubated with 200 µg/ml Alexa488-conjugated fibrinogen for 30 min at 37°C; washed; diluted ten-fold and analyzed by flow cytometry. Cells were also incubated with Alexa488-conjugated 10E5 to assess αIIbβ3 expression. Data are expressed as net, normalized fluorescent intensity (NNFI), calculated as described previously described ([28]insert ref 28 – Blue) using the geometric mean fluorescence intensity in the absence of priming and in the presence of unlabeled mAb 10E5 as background.

### Clot retraction

Cells were harvested with trypsin and EDTA, washed sequentially with culture medium and HBMT buffer without cations, and resuspended to 6 X 10^6^ cells/ml. A 0.5 ml aliquot of the cell suspension was placed in a 60 X 8 mm glass cuvette and then CaCl_2_ (5 mM final), fibrinogen (200 µg/ml final) and thrombin (2 U/ml final) were added and the cells were mixed. The cuvettes were maintained at 37°C and photographed at timed intervals for up to 4 hrs.

Biotinylation, immunoprecipitation, immunoblotting, soluble ligand binding (PAC-1, fibrinogen, AP5, and kistrin) and adhesion to collagen and immobilized fibrinogen were performed as previously described [16].

## Results

### TMD stimulation

**Figure 1:**
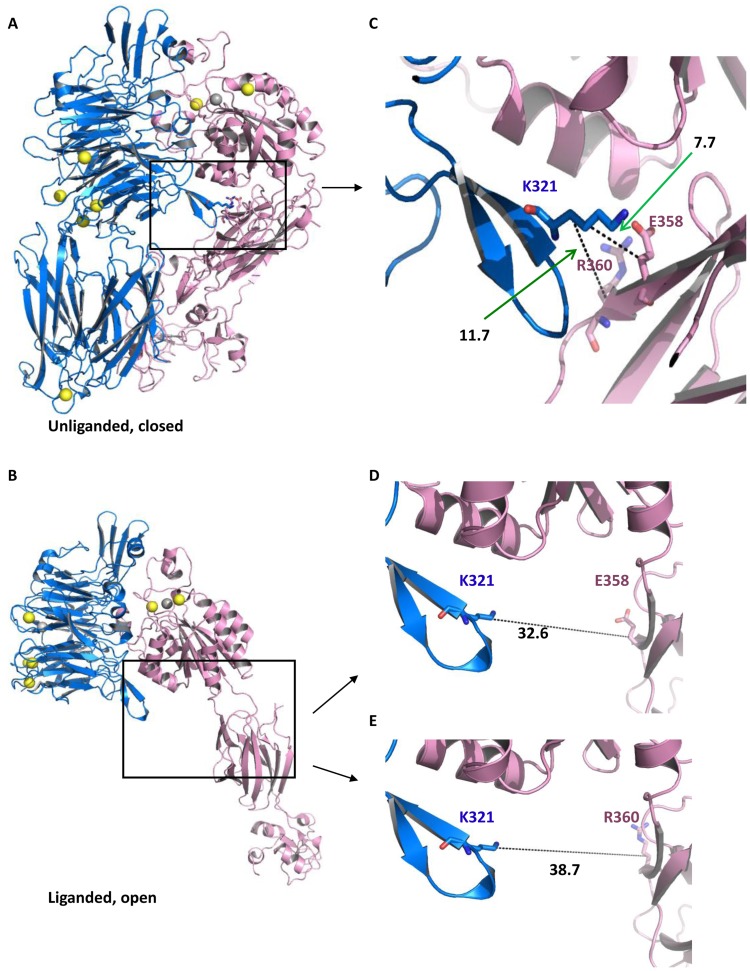
Structures of integrin αIIbβ3 in unliganded, closed and liganded, open conformations. αIIb subunit is in blue color, β3 subunit is in pink color. **A.** Unliganded, closed structure of αIIbβ3 (PDB: 3FCS) [7] **B.** Liganded, open structure of αIIbβ3 (PDB: 3FCU)[7]. **C.** Structural details of unliganded, closed αIIbβ3. The distances between the Cβ atoms of αIIbK321 and β3E358 and between αIIbK321 and β3R360 are 7.7 and 11.7 Å, respectively. **D** and **E.** Structural details of the liganded, open αIIbβ3. The distances between the Cβ atoms of αIIbK321and β3E358 and between αIIbK321and β3R360 are 32.6 and 38.7 Å, respectively.

In the unliganded, closed αIIbβ3 structure, αIIbK321 in the β-propeller domain and β3E358 in the hybrid domain form a salt bridge at the interface between the two subunits (PDB: 3FCS) [7] (Fig. 1A and C). Their Cβ atoms are 7.7 Å apart. In the liganded, open structure, in contrast, their Cβ atoms are 32.6 Å apart (Fig. 1B and D) (PDB: 3FCU) [7]. This salt bridge breaks very early in the swing-out motion and thus we reasoned that preventing these residues from separating would stop the earliest conformational changes associated with swing-out. Similarly, in the unliganded, closed αIIbβ3 structure, αIIbK321 is close to β3R360 in the hybrid domain, with the distance between Cβ atoms being 11.7 Å (PDB: 3FCS) (Fig. 1A and C). In the liganded structure, the Cβ atom distance increases to 38.7 Å (PDB: 3FCU) (Fig. 1B and E). As a result, we decided to make two different mutant receptors, αIIbK321C/β3E358C and αIIbK321C/β3R360C.

### Heterodimer disulfide bond formation in both XS-O mutants is supported by SDS-PAGE analysis and mass spectroscopy

**Figure 2:**
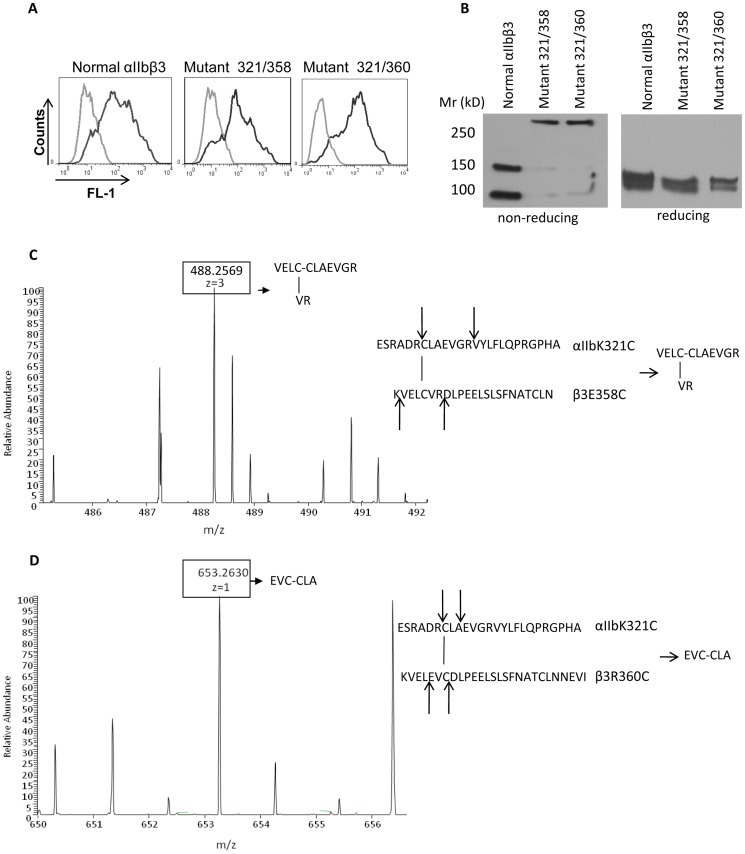
Analysis of mutant protein expression. A. Surface expression of αIIbβ3 on HEK293 cells expressing normal or mutant αIIbβ3 receptors as judged by the binding of mAb 10E5. 1 million cells in 100 µl were incubated with 5 µg/ml Alexa488-conjugated mAb 10E5 (black line) or as a control for non-specific binding, 5 µg/ml Alexa488-conjugated mAb 10E5 in the presence of excess (125 µg/ml) unlabelled 10E5 (gray line; background). **B. Disulfide-bonded αIIbβ3 heterodimer formation on the cell surface of XS-O mutants.** HEK293 cells expressing normal αIIbβ3 or XS-O mutants were biotinylated and lysed, and lysates were immunoprecipitated with anti-αIIbβ3 complex mAb 10E5. After SDS-PAGE and protein transfer, biotinylated proteins were identified with streptavidin-HRP. **Left panel.** Non-reduced SDS-PAGE analysis of normal αIIbβ3 and XS-O mutants. **Right panel.** SDS-PAGE analysis of normal αIIbβ3 and XS-O mutants under reducing conditions (10% β-mercaptoethanol). **C and D. Mass spectroscopy identifies unique, predicted peptides from XS-O mutants.** **C.** Purified protein from mutant 321/358 was digested with trypsin and analyzed by LC-MS/MS. A peak at m/z = 488.2569 corresponded to the predicted disulfide linked MH_4_^3+^ ion of peptide VELC(-VR)-CLAEVGR. **D.** Purified protein from mutant 321/360 was digested with trypsin and ASP-N, followed by LC-MS/MS analysis. A peak at m/z = 653.2639 corresponded to the predicted disulfide linked MH_4_^+^ ion of EVC-CLA.

As judged by the binding of anti-αIIbβ3 mAb 10E5, αIIbβ3 expression was similar on cells expressing the XS-O mutants and cells expressing normal αIIbβ3 (Fig. 2A). To assess whether the mutant proteins contained the expected disulfide bonds, we immunoprecipitated the biotin surface-labeled proteins and then analyzed them by SDS-PAGE and streptavidin staining. αIIb (with an Mr of 140 kD) and β3 (with an Mr of 95 kD) were immunoprecipitated with anti-αIIbβ3 complex-specific mAb 10E5 from normal αIIbβ3 transfected HEK293 cells (Fig. 2B). In contrast, an additional band of Mr ∼250 kD, corresponding to the Mr expected if αIIb is covalently coupled to β3 was observed in samples of the two XS-O mutants. To assess whether the Mr 250 kD band contained both αIIb and β3, we performed immunoblotting with mAbs specific for each subunit. Both mAb PMI-1 (anti-αIIb) and 7H2 (anti-β3) reacted with the 250 kD band (Fig. S2), demonstrating the presence of both subunits. As expected, with reduction (Fig. 2B), the normal αIIbβ3 showed a decrease in Mr of αIIb and an increase in Mr of β3. With both mutants, reduction led to both complete loss of the Mr 250 kD band, indicating that it depended on disulfide cross-linking, and enhanced intensity of the αIIb and β3 subunits. The effects of different concentrations of DTT and different times of incubation on the XS-O mutants were assessed by immunoblotting with the αIIb-specific mAb PMI-1 (Fig. S3).

Mass spectroscopy of a trypsin digest of mutant 321/358 demonstrated a peak in the main spectrum at m/z = 488.2569, which corresponds to the m/z expected from the unique peptide resulting from a disulfide linkage between the peptides VELCVR and CLAEVGR (MH_4_^3+^) predicted from the creation of the engineered disulfide bond (Fig. 2C). Since the 321/360 mutant converted the trypsin cleavage site amino acid R360 to C, an alternative cleavage strategy was employed to assess this mutant, combining trypsin and Asp-N. A peptide was identified in the main spectrum at m/z = 653.2630 corresponding to the unique disulfide bonded peptide EVC-CLA (MH_4_^+^) predicted from the creation of the engineered disulfide bond in mutant 321/360 (Fig. 2D). The identities of these two peptides were confirmed by tandem mass spectrometry. Neither peptide was present in normal αIIbβ3.

### XS-O mutants 321/358 and 321/360 have impaired ability to bind the high Mr ligand-mimetic mAb PAC-1 and the high Mr ligand fibrinogen; Introduction of the activating mutations αIIbF992A/F993A into the XS-O mutants does not rescue binding of PAC-1 or fibrinogen

**Figure 3:**
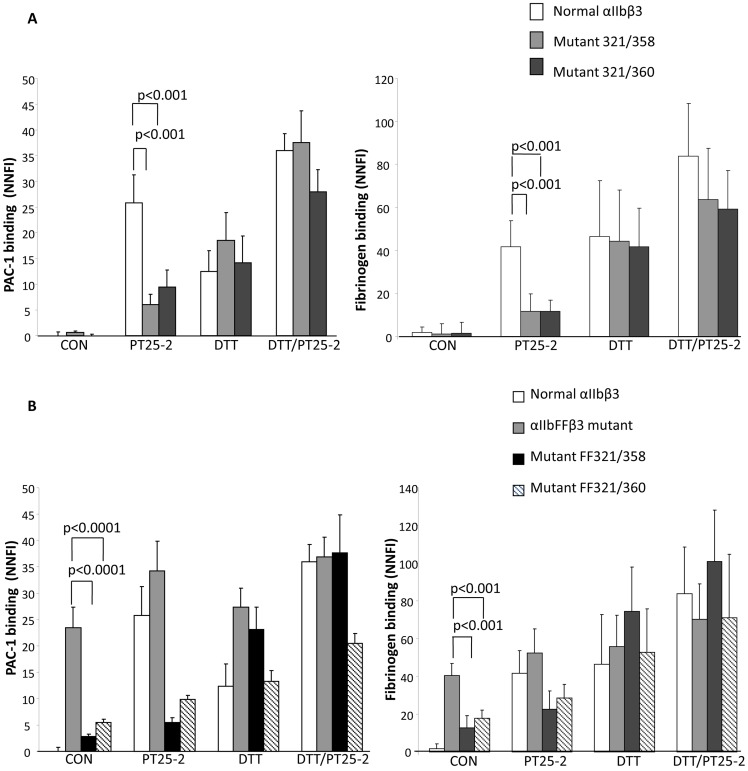
Cells expressing XS-O mutants have reduced ability to bind PAC-1 and fibrinogen. **A. Left**, PAC-1 binding of cells expressing XS-O mutants 321/358 and 321/360 the absence of treatment (control; CON) or in the presence of mAb PT25-2, DTT, or DTT+PT25-2. FITC-PAC-1 was added at 5 µg/ml and binding was assessed via flow cytometry. Binding is expressed as net normalized fluorescence intensity (NNFI), in which the geometric mean fluorescence intensity after subtracting nonspecific binding is divided by the relative surface receptor expression as judged by the binding of mAb 10E5. Data expressed as mean ± SD; n = 5. **Right**, Fibrinogen binding using Alexa488-fibringen (200 µg/ml) as indicated above in “**A**” for PAC-1 (mean ± SD; n = 6). **B.** The αIIb F992A/F993A activating mutations fail to rescue PAC-1 and fibrinogen binding in the XS-O mutants.

Cells expressing normal αIIbβ3 did not bind PAC-1 in the absence of activation (Fig. 3A, left). In the presence of the activating mAb PT25-2, PAC-1 binding to the cells expressing normal αIIbβ3 increased from an NNFI of 0±0.8 to 25.8±5.4. Both the 321/358 and 321/360 mutants also bound little PAC-1 in the absence of PT25-2 (NNFI = 0.5±0.4 and 0±0.4, n = 5). In contrast to normal αIIbβ3, however, both mutants bound significantly less PAC-1 in the presence of PT25-2 (321/358: NNFI = 5.9±1.9, n = 5, p<0.001; 321/360: NNFI = 9.4±3.3, n = 5, p<0.001). DTT treatment partially or completely rescued the ligand binding ability of the XS-O mutants. Data with fibrinogen binding were similar to those with PAC-1 binding (Fig. 3A, right).

The αIIbF992A/F993Aβ3 mutant (αIIbFFβ3) has been reported to be constitutively active as a result of the mutations disrupting the association of the membrane-proximal portions of the α and β subunit cytoplasmic domains [31]–[33]. Similar disruption of the α and β subunit cytoplasmic domains is proposed to occur with inside-out activation of the receptor [34]. To assess whether the αIIbFF mutations could rescue the XS-O mutants' ability to bind high Mr ligands, we co-expressed the αIIbFF mutant with normal β3 and the αIIb mutant K321C, F992A, F993A with either the β3E358C or the β3R360C mutant. Immunoblot analysis of the surface membrane-labeled receptors demonstrated that cross-linked heterodimers containing the mutant αIIb and β3 chains were expressed on the cell surface and could be immunoprecipitated by mAb 10E5 (Fig. S4).

Consistent with our previous findings, there was little PAC-1 binding to cells expressing normal αIIbβ3 in the absence of stimulation. In sharp contrast, PAC-1 binding to the αIIbFFβ3 mutant in the absence of PT25-2 was comparable to that of cells expressing normal αIIbβ3 in the presence of PT25-2 (NNFI: 23.4±3.9 *vs* 25.8±5.4) (Fig. 3B left). In the absence of stimulation, cells expressing both the αIIbFF mutations and the XS-O mutations bound somewhat more PAC-1 than did the normal αIIbβ3, but much less than the αIIbFFβ3 mutant. Adding PT25-2 greatly enhanced PAC-1 binding to normal αIIbβ3, but had only modest impact on PAC-1 binding to the αIIbFFβ3 mutant or the combined mutants. DTT treatment partially or completely rescued the ligand binding ability of the combined mutants. The fibrinogen binding results paralleled the PAC-1 binding results (Fig. 3B right).

### Kistrin-induced AP5 binding to XS-O mutants 321/358 and 321/360 is less than to normal αIIbβ3 or the FF321/358 and FF321/360 mutants

**Figure 4:**
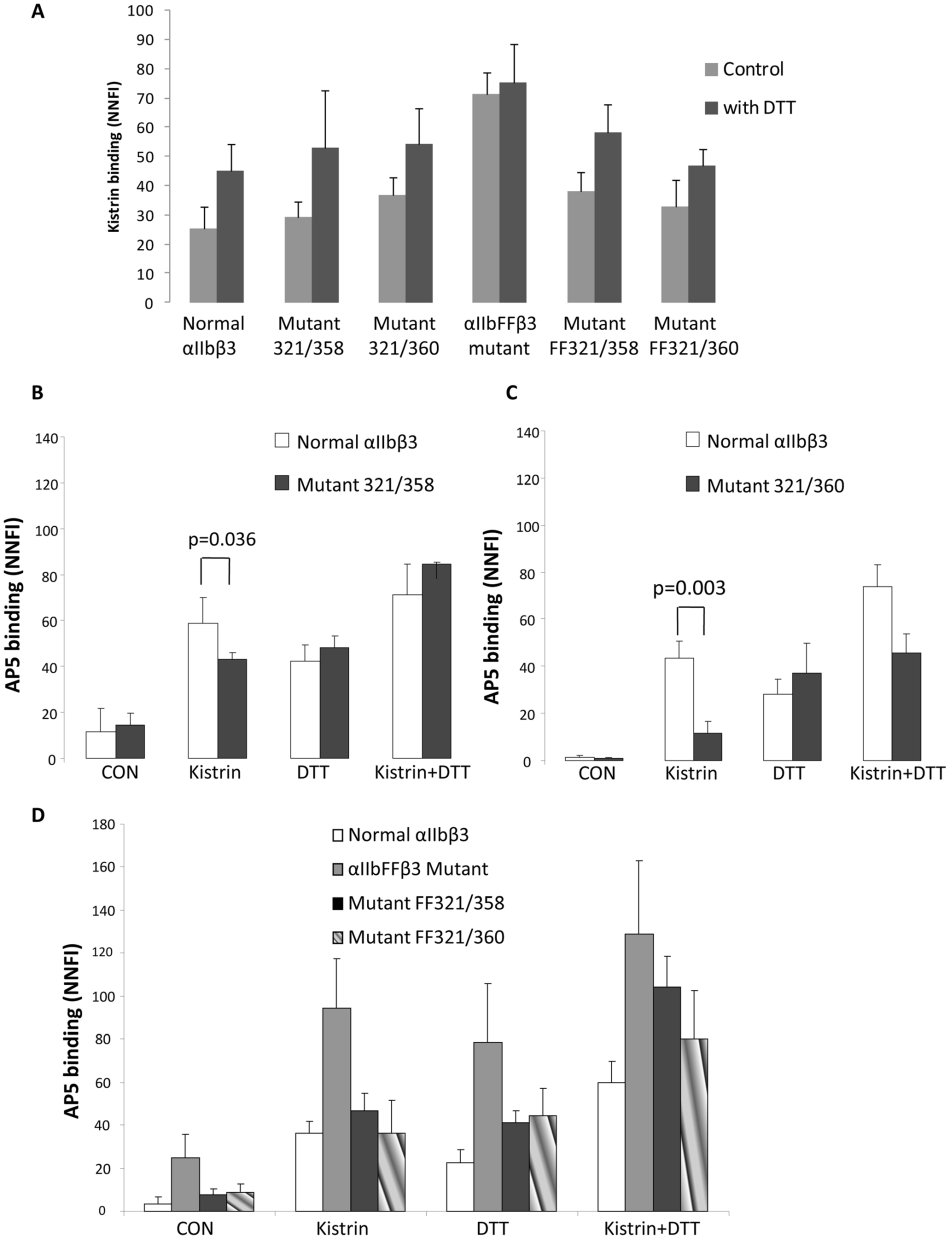
Kistrin-induced AP5 binding to cells expressing XS-O mutants. A. Kistrin binds to cells expressing XS-O mutants. Cells expressing normal αIIbβ3 and XS-O mutants were either untreated or treated with DTT (5 mM) before being incubated with Alexa488-kistrin (50 nM). Binding was assessed via flow cytometry and expressed as net normalized fluorescence intensity (mean ± SD; n = 4). **B. AP5 binding to mutant 321/358.** Alexa488-labeled anti-LIBS mAb AP5 was incubated with cells in the absence or presence of kistrin (200 nM), or DTT (5 mM), or both at 37°C for 1 h. AP5 binding was assessed by flow cytometry and expressed as NNFI, using excess unlabeled AP5 to assess nonspecific binding. **C. AP5 binding to XS-O mutant 321/360. D. AP5 binding to αIIbFFβ3 mutant and mutants FF321/358 and FF321/360** (mean ± SD; n = 4).

Kistrin is a small RGD-containing snake venom protein. In the absence of DTT, kistrin bound similarly to normal αIIbβ3, both XS-O mutants, and both FFXS-O mutants; it bound at higher levels, however, to the αIIbFFβ3 mutant (P<0.001, n = 4) (Fig. 4A). DTT treatment enhanced kistrin binding to normal αIIbβ3 and the four XS-O mutants, but the percentage increase was much less than with PAC-1 or fibrinogen binding (Fig. 3).

Since XS-O mutants can bind kistrin similarly to normal αIIbβ3, we further evaluated anti-LIBS AP5 binding induced by kistrin. Relatively little AP5 bound to normal αIIbβ3 or the XS-O mutant 321/358 in the absence of activation. AP5 binding increased to both receptors in the presence of kistrin, DTT, and kistrin+DTT (Fig. 4B). Kistrin produced a greater effect, however, on normal αIIbβ3 than on the 321/358 mutant (p = 0.036, n = 3). A similar pattern was found with the 321/360 mutant (Fig. 4C), but this mutant bound significantly less AP5 than normal αIIbβ3 (p = 0.003, n = 3) or the 321/358 mutant (p = 0.001, n = 3) in the presence of kistrin. DTT treatment partially or completely rescued the AP5 binding of the XS-O mutants.

In the absence of activation, αIIbFFβ3 bound significantly more AP5 than did normal αIIbβ3 (p = 0.003, n = 5) or either XS-O mutant (p = 0.01 and 0.015 respectively) (Fig. 4D). Adding kistrin or DTT increased the binding of AP5 to all of the receptors, but the αIIbFFβ3 mutant again bound more than any of the other receptors. Combining kistrin and DTT further increased binding to all of the receptors, but the αIIbFFβ3 mutant still bound the most.

### Mutant 321/358 and 321/360 can adhere to immobilized fibrinogen, adhesion of XS-O mutant 321/358 to immobilized fibrinogen is mediated by the covalently-associated heterodimer

**Figure 5:**
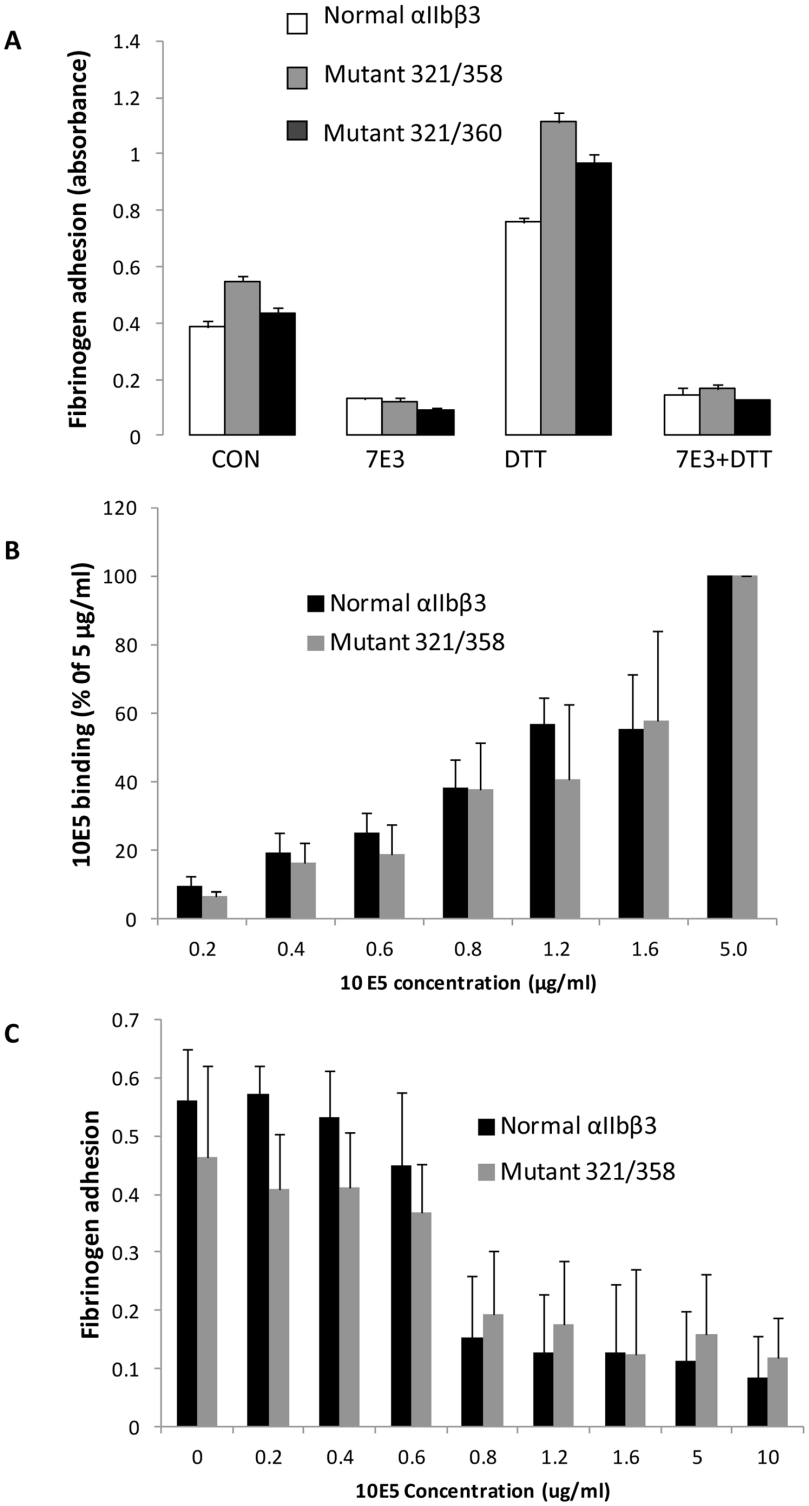
Cells expressing mutant 321/358 and 321/360 can adhere to immobilized fibrinogen. **A.** Cells expressing normal αIIbβ3 or XS-O mutants 321/358 and 321/360 were allowed to adhere to microtiter wells coated with 5 µg/ml fibrinogen without treatment or in the presence of mAb 7E3, DTT, or 7E3+DTT. Adhesion was measured by lysing the washed, adherent cells and assaying for acid phosphatase activity (mean ± SD, n = 4). Relative surface expression of αIIbβ3 based on mAb 10E5 binding in these experiments was 80% for normal αIIbβ3, 100% for 321/358, and 90% for 321/360. **B. Binding of mAb 10E5 to cells expressing normal αIIbβ3 or XS-O mutant 321/358.** Data are expressed as percentage of the value at 5 µg/ml 10E5. The GMFI values for 10E5 binding at 5 µg/ml were 75±14 for the cells expressing normal αIIbβ3 and 129±39 for the cells expressing the 321/358 mutant (n = 4). **C. Adhesion to fibrinogen of cells expressing normal αIIbβ3 or XS-O mutants 321/358 in the presence of different concentrations of mAb 10E5.** The same cells used in **B**. were tested for adhesion to fibrinogen (n = 4).

Cells expressing αIIbβ3 receptors can adhere to fibrinogen-coated surfaces in the absence of exogenous activation [35], [36]. Cells expressing both XS-O mutants adhered to fibrinogen immobilized at low density (5 µg/ml) slightly better than cells expressing normal αIIbβ3 (Fig. 5A). These small differences paralleled, and thus were probably caused by, the minor differences in receptor surface expression (normal αIIbβ3∶321/358∶321/360 = 80∶100∶90). The mAb 7E3 inhibited adhesion of all three cell lines to similar extents. DTT enhanced the adhesion of all three cell types and 7E3 was able to inhibit the increased adhesion in the presence of DTT. Similar data were obtained when fibrinogen was immobilized at 50 µg/ml (data not shown).

To assess whether the adhesion to fibrinogen by the XS-O mutant 321/358 was mediated by the relatively small percentage of αIIbβ3 receptors that may not have undergone disulfide bond formation, we repeated the adhesion experiments with dose-response inhibition of adhesion by mAb 10E5. Both the cells expressing normal αIIbβ3 and the 321/358 mutant showed similar binding curves for mAb 10E5 when tested at multiple concentrations (Fig. 5B). In the absence of mAb 10E5, the adhesion of cells expressing normal αIIbβ3 and the 321/358 mutant to immobilized fibrinogen were similar as judged by the absorbance values (Fig. 5C). We reasoned that if the cells expressing the 321/358 mutant adhered to fibrinogen via the ∼10–15% of the receptors that did not undergo heterodimer formation (Fig. 2B), they would be more sensitive to the inhibitory effects of mAb 10E5. In fact, however, the mAb 10E5 dose-response inhibition of adhesion of the cells expressing the 321/358 mutant was very similar to the dose-response inhibition for the cells expressing normal αIIbβ3 (Fig. 5C). To assess whether αVβ3 contributed to the adhesion, we also performed the experiments in the presence of mAb LM609 and found no effect on the adhesion of cells expressing normal αIIbβ3 and less than a 4% decrease in the adhesion of the cells expressing the 321/358 mutant (data not shown). These data support the conclusion that covalently bonded mutant receptors mediated adhesion to fibrinogen.

### Cells expressing the XS-O mutants 321/358 and 321/360 have defective cytoskeletal reorganization after adhering to immobilized fibrinogen

**Figure 6:**
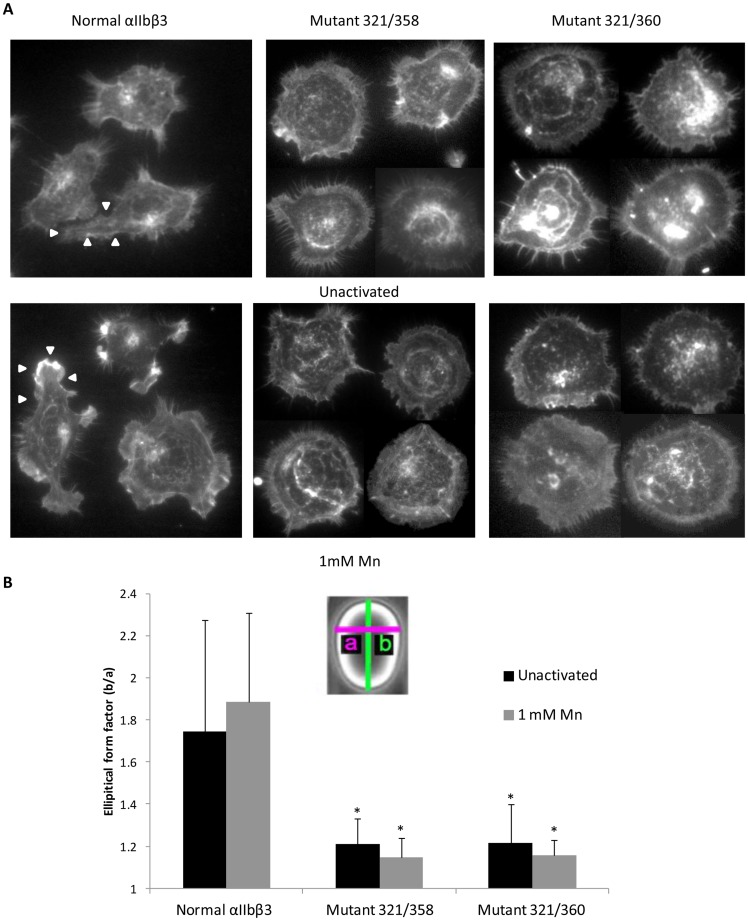
Cells expressing XS-O mutants 321/358 and 321/360 have defective cytoskeletal reorganization on immobilized fibrinogen. **A.** Cells expressing normal αIIbβ3 or XS-O mutants 321/358 or 321/360 were allowed to adhere to microtiter wells coated with 20 µg/ml fibrinogen for 2 hr in HBMT buffer with 2 mM Ca^2+^ and 1 mM Mg^2+^. Cells were fixed, permeabilized, and double-stained for actin filaments with rhodamine-phalloidin (red) and β3 with anti-β3 antibody Alex 488-7H2 (green). Image J was used to merge the two color images. **Upper panel.** Untreated cells. **Lower panel.** Cells treated with 1 mM Mn^2+^. **B.** The eccentricity of cell shape was measured by fitting an ellipse into the image of the cell and measuring both the major and minor axes. Eccentricity was defined as the ratio of the major axis (**b**) to the minor axis (**a**) and expressed as the elliptical form factor. * p<0.0001 compared to normal αIIbβ3 (n = 20).

After adhering to fibrinogen, cells expressing normal αIIbβ3 formed filopodia and lamellipodia, reorganized their actin into filaments detectable with phalloidin and became eccentric in shape (Fig. 6A upper). In contrast, the adherent cells expressing either the XS-O 321/358 or 321/360 mutant were nearly circular, with some irregular, short filopodia. When Mn^2+^ was used to activate the cells, cells expressing normal αIIbβ3 demonstrated increased cytoskeletal reorganization, forming more lamellipodia and adopting a more irregular shape. Cells expressing the XS-O mutants, however, did not demonstrate enhanced cytoskeletal reorganization in the presence of Mn^2+^ (Fig. 6A lower). Cells expressing normal αIIbβ3 formed multiple focal adhesions as shown by the colocalization of vinculin and actin filaments and the colocalization of β3 and actin filaments (Figs. S5 and S6). In both cases, the images show organized actin filaments connecting focal adhesions in the filopodia. In contrast, the mutant cells show a distinctive round shape with no concentration of vinculin in focal adhesions. Moreover, the actin filaments are arrayed radially and in loops at the periphery of the cell rather than spanning between focal adhesions.

We also conducted time-lapse photography of the adhesion and spreading of cells expressing normal or mutant αIIbβ3 using differential interference microscopy (Videos S1, S2, S3, S4) and the results demonstrated that the cells expressing normal αIIbβ3 adhere and spread with a discrete change in morphology at about 15 min as they begin to extend filopodia and lamellipodia and reorganize their cytoskeletons. In contrast, the cells expressing the mutant receptor maintain their round shape throughout and undergo repeated radial extension of the membrane, but without the development of mature, organized filopodia.

Quantitative analysis of cell area showed no differences between the cells expressing normal and the XS-O mutants (data not shown), but the cells expressing normal αIIbβ3 spread in a more eccentric manner as judged by the elliptical form factor, reflecting differences in developing lamellipodia and focal adhesions (Fig. 6B, p<0.0001 for both mutants). As a control, cells expressing normal αIIbβ3 or XS-O 321/358 were analyzed for their adhesion, spreading, and cytoskeletal reorganization on collagen (data not shown). Both cell types spread equally well and showed similar elliptical form factors (1.66±0.26 and 1.64±0.33; p = 0.78). Thus, the morphologic abnormalities found with adhesion to fibrinogen are not due to a generalized defect in cytoskeletal reorganization, but rather appear to be specific for the αIIbβ3 mutations.

### XS-O mutant 321/358 have reduced fibrinogen binding after priming

**Figure 7:**
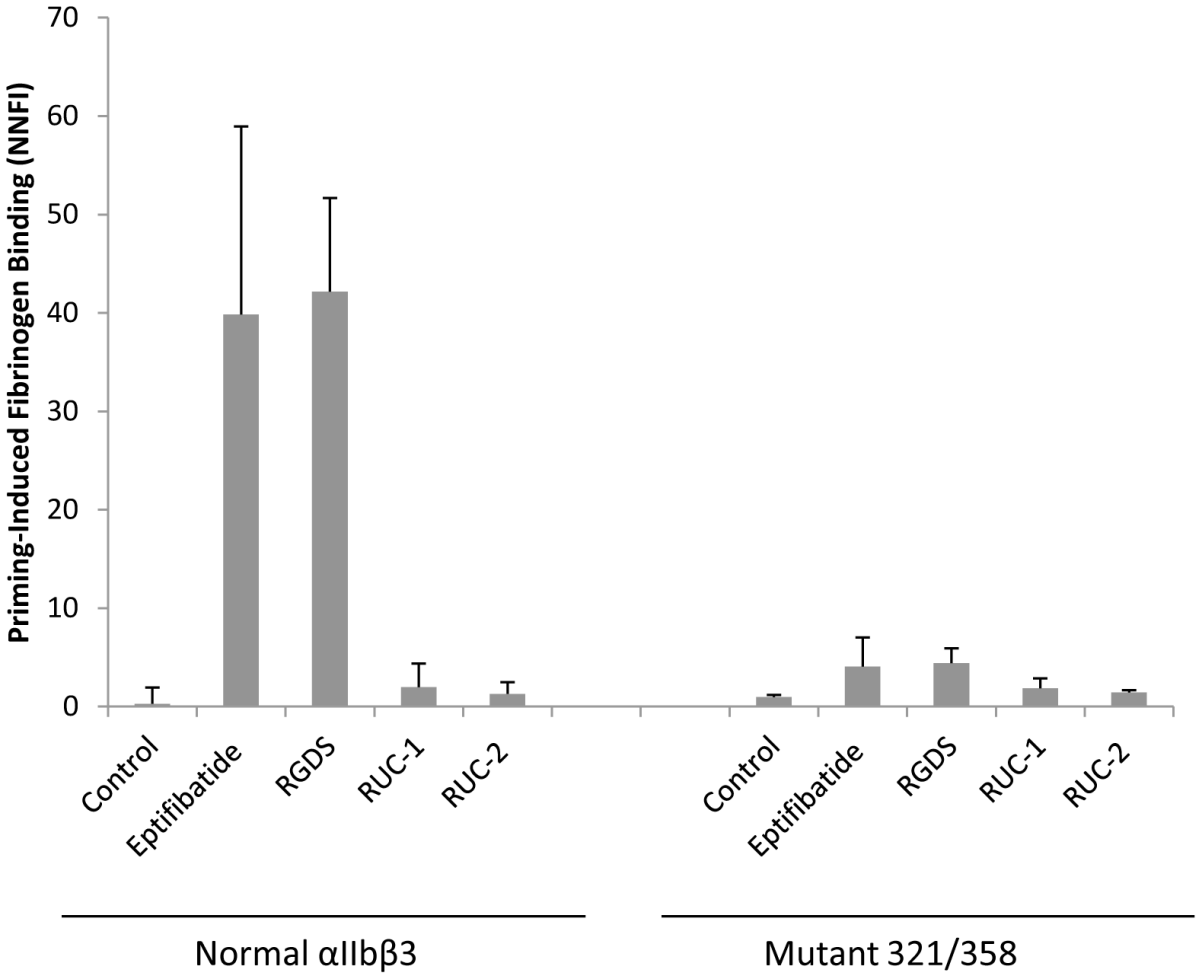
Fibrinogen binding of mutant 321/358 after priming. HEK293 cells expressing normal αIIbβ3 or mutant 321/358 were incubated with buffer (Control), eptifibatide (1 µM), RGDS (100 µM), RUC-1 (100 µM), RUC-2, (1 µM), or EDTA (10 mM) for 20 min at RT and then fixed and washed 4 times. Binding of Alexa488-conjugated fibrinogen (200 µg/ml) was then assessed by flow cytometry and expressed as NNFI, in which the net geometric mean fluorescence intensity after subtracting the background is divided by the relative surface receptor expression as judged by the binding of mAb 10E5. Data expressed as mean ± SD after subtracting the EDTA value as background (n = 3).

Cells expressing normal αIIbβ3 bound little fibrinogen, but pre-incubating the cells with eptifibatide or the peptide RGDS primed the cells to bind fibrinogen (P = 0.02 and p = 0.001 respectively, n = 3) (Fig. 7). In contrast, the cells expressing the 321/358 mutant bound significantly less fibrinogen in the presence of each priming agent (n = 3; p = 0.03 for eptifibatide and p = 0.002 for RGDS). The small molecule αIIbβ3 antagonists RUC-1 and RUC-2 served as controls since they both bind to αIIbβ3, but do not induce fibrinogen binding [28]–[30]. To assess whether eptifibatide bound to the 321/358 mutant, we compared the ability of eptifibatide to inhibit the adhesion of cells expressing normal αIIbβ3 or the 321/358 mutant to fibrinogen. The dose response was similar for both cell lines, with 3 µM eptifibatide inhibiting normal αIIbβ3 adhesion by 62±11% (n = 4) and the 321/358 mutant by 83±10% (n = 4). At 10 µM eptifibatide the values were 91±9% and 99±3%, respectively. The RGDS compound could not be tested in the same manner because it did not inhibit adhesion of the normal αIIbβ3 cells at 300 µM, presumably reflecting its lower affinity.

### Cells expressing the XS-O mutant 321/358 can retract fibrin clots

**Figure 8:**
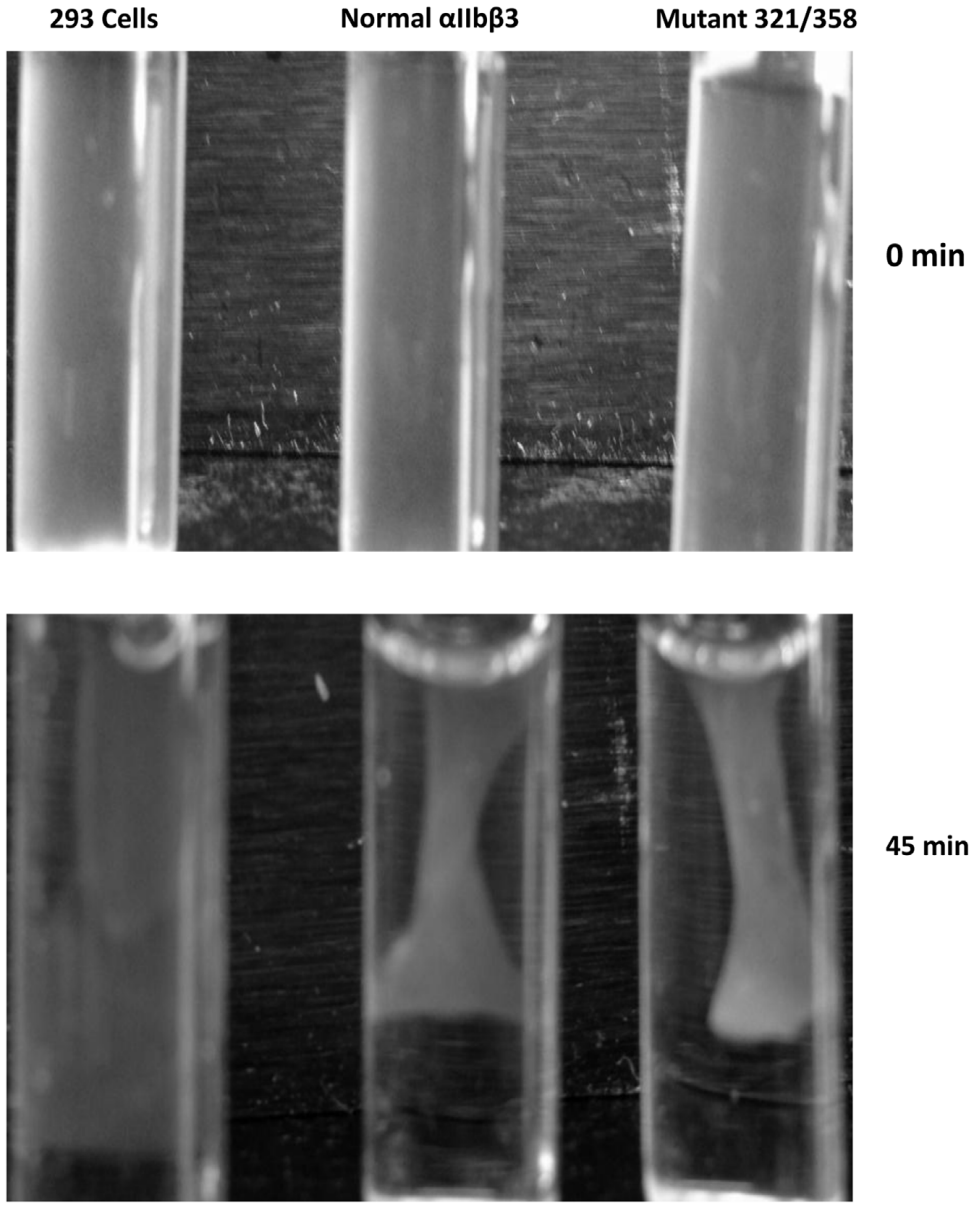
Cells expressing XS-O mutant 321/358 can retract fibrin clots. Untransduced cells (control 293 cells), cells expressing normal αIIbβ, and cells expressing XS-O mutant 321/358 were incubated with 200 µg/ml fibrinogen and 2 U/ml thrombin at 37°C. Clot retraction was monitored by photography at timed intervals up to 4 hr. The 45 min time point is shown since maximal clot retraction was achieved at this time.

Untransfected cells showed minimal clot retraction, but cells expressing both normal and XS-O 321/358 were able to retract fibrin clots to the same extent and at the same rate as judged by serial photographs over time (Fig. 8).

## Discussion

Integrin β3 hybrid domain swing-out is closely associated with integrin activation and the adoption of the high affinity ligand binding state [8], [9], [15], but the precise contribution of swing-out to ligand binding and the sequence of events remain unclear. Using data from TMD simulations, we identified the αIIb β-propeller residue K321 and the β3 hybrid domain residues E358 and R360 as candidates for cysteine mutagenesis to create new disulfide bonds to restrict the swing-out motion [19]. The creation of the expected αIIb321-β3358 and αIIb321-β3360 heterodimers was confirmed by SDS-PAGE and mass spectroscopy. Binding of both activation-dependent high Mr ligands (PAC-1 and fibrinogen) to the XS-O 321/358 and 321/360 mutants in the presence of PT25-2 is significantly reduced when compared to high Mr ligand binding to normal αIIbβ3. The residual ligand binding to the XS-O mutants might reflect incomplete disulfide bond formation or relatively low affinity binding to the mutant receptor. We conclude that limiting β3 swing-out prevents the receptor from adopting the conformation(s) required to bind activation-dependent high Mr soluble ligands.

In contrast to the data with the activation-dependent high Mr ligands, the XS-O mutants are able to bind the lower Mr snake venom protein kistrin. Thus, swing-out is not required for the binding of this ligand, which may reflect its smaller size and/or higher affinity. Kistrin binding to the XS-O mutants fails, however, to fully expose the AP5 binding site, indicating that the exposure of the AP5 binding site requires some contribution from swing-out. Similarly, the αIIb activating mutations increased exposure of the AP5 binding site on normal αIIbβ3, whereas it produced less increase in AP5 binding to the XS-O mutants, supporting a role for swing-out in the exposure of the AP5 epitope. XO-mutant 321/360 bound much less AP5 than mutant 321/358 in the presence of kistrin, possibly because 321/360 adopts a more compact conformation than 321/358, which is perhaps reflected in the fact that XO-mutant 321/360 is more resistant to DTT treatment (Fig. S3). Collectively these data are similar to those we obtained with an αIIb mutant designed to prevent extension of the αIIb subunit around the genu [16]. The defect in soluble ligand binding to that mutant could, however, be overcome by introducing an activating mutation in the β3 β I domain thought to induce swing-out. This raises the possibility that the major impact of receptor extension is to facilitate swing-out, perhaps by diminishing restrictive headpiece-tailpiece interactions. In this model, swing-out is downstream from extension and proximate to ligand binding.

αIIbβ3 activation and ligand binding in platelets is thought to be initiated by inside-out signaling, leading to separation of the αIIb and β3 cytoplasmic and transmembrane domains, and terminating in conformational changes in the head region that lead to extension and swing-out. To simulate this process in the HEK293 cell line, we introduced the F992A/F993A mutations into αIIb to produce a constitutively active receptor [33], [37], [38]. When combined with normal β3, the αIIbFFβ3 mutant receptor binds PAC-1 and fibrinogen constitutively. Combining the F992A/F993A mutations with the XS-O 321/358 or 321/360 mutations does not, however, rescue the XS-O mutants' ability to bind PAC-1 or fibrinogen, either constitutively or in the presence of PT25-2. If the αIIb F992A/F993A mutations do induce receptor extension, these data are consistent with the above model in which the swing-out motion is required for the binding of select activation-dependent high Mr ligands and is downstream from both αIIbβ3 leg separation induced by inside-out signaling and the conformational change(s) induced by the binding of mAb PT25-2 to the αIIb β-propeller domain. Alternatively or additionally, β3 subunit extension may provide large soluble ligands greater access to the ligand binding pocket.

Low molecular weight ligands patterned after the RGD sequence and RGD-containing peptides can “prime” the αIIbβ3 receptor such that after washing away the free low molecular weight ligand the receptor can bind fibrinogen [28], [30], [39]–[41]. This activation of the receptor has been hypothesized to contribute to the paradoxical increase in deaths ascribed to several oral αIIbβ3 antagonists patterned after the RGD sequence [42], [43]. To assess whether “priming” requires β3 integrin subunit swing-out, we tested the priming effect of eptifibatide and the peptide RGDS on cells expressing normal αIIbβ3 and the XS-O 321/358 mutant. We found that the mutant receptor had a markedly reduced ability to bind soluble fibrinogen after priming. Thus, it appears that receptor priming requires β3 swing-out, raising the possibility that therapeutic agents do not induce swing-out may have a reduced risk of paradoxical receptor activation [28], [30], [42], [43].

Our data on soluble fibrinogen, PAC-1, and LIBS mAb binding to the XS-O mutants are similar to those reported by Luo et al. with αIIbβ3 containing a β3T329C/A347C double mutation designed to prevent the motion of the β I domain α7 helix associated with β3 swing-out [14]. Their mutant differs from ours, however, in being unable to support cell adhesion to immobilized fibrinogen. Since their mutation introduces constraints within the β I domain whereas ours primarily constrains the movement of the hybrid domain away from the β I domain, intra-β I conformational changes may be required for binding immobilized fibrinogen.

While our studies were in progress, Kamata et al. reported the effect of creating cysteine mutations in αIIbD319 and β3V359 to create a disulfide bond similar to ours to prevent swing-out of the hybrid domain [17]. Their mutant, like ours, did not bind soluble fibrinogen in the presence of the activating mAb PT25-2. This defect in ligand binding could not be overcome by introducing mutations into the β3 subunit (Q595N/R597T) designed to induce receptor extension. Thus, their data also support the hypothesis that swing-out is downstream from receptor extension. They concluded that swing-out is required for “high affinity ligand binding,” but we would temper this conclusion by restricting the requirement for swing-out to the binding of select activation-dependent high Mr soluble ligands. For example, as in our studies, they found that the inability to bind soluble ligands was selective, since the mAb OP-G2 was able to bind to the mutant. Moreover, the small GRGDS peptide was presumed to bind to their 319/359 mutant receptor.

Our data on cell adhesion and cytoskeletal reorganization extend those of Kamata et al. by examining the effect of limiting the swing-out motion on the interaction of αIIbβ3 with immobilized ligand. Of particular note, we found that the XS-O mutant receptors are capable of supporting cell adhesion to immobilized fibrinogen. These data also echo those we obtained with the αIIb mutant designed to prevent extension about the αIIb genu [16], reinforcing that inhibiting extension and swing-out produce similar functional defects. Potential explanations for the XS-O mutant's ability to support cell adhesion to immobilized fibrinogen include: 1. immobilizing fibrinogen at high density increases receptor avidity enough to compensate for decreased affinity [35], 2. immobilizing fibrinogen alters its conformation so that it can: a) bind with high affinity even without β3 swing-out, b) bind to another site on αIIbβ3, or c) bind to another receptor [44]. To address the first possibility, we used two different concentrations of fibrinogen, including one designed to be nearly limiting in density [45], and did not observe a difference in the relative cell adhesion by the mutants. It appears unlikely that the immobilized fibrinogen is binding to another site on αIIbβ3 or to another receptor since both eptifibatide and the mAb 7E3 inhibited the adhesion of cells expressing normal αIIbβ3 or the XS-O mutants.

We also found that the mutant receptors are unable to support normal outside-in signaling required for normal cytoskeletal reorganization on immobilized fibrinogen via lamellipodia and the formation of focal adhesions. Thus, β3 hybrid domain swing-out is required for the outside-in signaling that results in cytoskeletal rearrangements [46].

In conclusion, our study demonstrates that β3 hybrid swing-out is necessary for the activation-dependent binding of select high Mr ligands to αIIbβ3, but not for the binding of αIIbβ3 to immobilized fibrinogen. It is likely that swing-out is downstream from receptor extension since interventions designed to initiate extension cannot rescue ligand binding to receptors that have limited ability to undergo swing-out. Finally, swing-out is necessary for receptor priming by low molecular weight ligands and integrin-mediated outside-in signaling, the latter suggesting that the swing-out motion transmits information to the cytoplasmic domain(s) of one or both subunits. The precise structural changes that connect swing-out to changes in ligand affinity and outside-in signaling and the precise sequence of events remain to be defined.

## Supporting Information

Figure S1 **HEK293 cells expressing normal αIIbβ3 express little or no αVβ3.** HEK293 cells expressing either normal αIIbβ3 or αVβ3 were lysed with Triton X-100 and the proteins in the lysates were resolved on SDS-PAGE. Integrin subunits were immunoblotted with anti-αIIb mAb PMI-1, an anti-αV antibody, or anti-β3 mAb 7H2.
(TIF)

Figure S2 **Disulfide-bonded heterodimers from XS-O mutants contain both αIIb and β3.** **A**. Lysates of HEK293 cells expressing XS-O mutants 321/358 or 321/360 were immunoprecipitated with mAb 10E5 followed by SDS-PAGE and immunoblotting with anti-αIIb mAb PMI-1. **B**. Cells were treated as in A, immunoprecipitated with anti-αIIb mAb Hip8 and immunoblotted with anti-β3 mAb 7H2. The 150 kD bands are the immunoglobulins used for the immunoprecipitations. The Mr 250 kD band stained positive with both PMI-1 and 7H2, indicating the presence of both αIIb and β3.
(TIF)

Figure S3 **The effects of DTT on XS-O mutants.** HEK293 cells expressing normal αIIbβ3 or XS-O mutants 321/358 and 321/360 were either untreated or treated with different concentration of DTT at 37°C for the indicated times. Cells were lysed with Triton X-100 after DTT treatment and proteins were separated by SDS-PAGE and immunoblotted with anti-αIIb antibody PMI-1. The 321/358 mutant cross-linked heterodimer showed marked dissociation when reacted with 3 mM DTT for 5 min and virtually complete dissociation with 5 mM DTT for 5 min. In contrast, the 321/360 demonstrated only minimal dissociation with 3 mM DTT and moderate dissociation with 5 mM for 5 min. Increasing the DTT concentration to 30 mM and lengthening the time of incubation to 30 min were required to achieve complete dissociation.
(TIF)

Figure S4 **Heterodimer formation of mutant FF321/358 and FF 321/360.** HEK293 cells expressing normal αIIbβ3 or the mutant receptors were biotinylated and lysed, and then the lysates were immunoprecipitated with anti-complex mAb 10E5 and analyzed by SDS-PAGE followed by staining with Streptavidin. Heterodimers containing the mutant αIIb and β3 chains were expressed on the cell surface and could be immunoprecipitated by mAb 10E5.
(TIF)

Figure S5 **Cells expressing XS-O mutant do not form focal adhesions after spreading on immobilized fibrinogen for 60 min.** Fluorescence microscopy of cells expressing normal αIIbβ3 or the XS-O mutant 321/358 stained with TRITC-phalloidin (F-actin; red) and FITC-anti-vinculin (focal contacts; green).
(TIF)

Figure S6 **Spreading of cells expressing normal αIIbβ3 or XS-O mutants 321/358 on fibrinogen analyzed by double-labeling β3 with an anti-β3 antibody (Alex 488-7H2) (green) and actin with TRITC-phalloidin (red).**
(TIF)

Video S1 **Spreading of cells expressing normal αIIbβ3 on fibrinogen.**
(AVI)

Video S2 **Spreading of cells expressing normal αIIbβ3 on fibrinogen.**
(AVI)

Video S3 **Spreading of cells expressing XS-O mutant 321/358 on fibrinogen.**
(AVI)

Video S4 **Spreading of cells expressing XS-O mutant 321/358 on fibrinogen.**
(AVI)
