# Supplementary figures and images for: Swing-Out of the β3 Hybrid Domain Is Required for αIIbβ3 Priming and Normal Cytoskeletal Reorganization, but Not Adhesion to Immobilized Fibrinogen
Source: PLoS One. 2013 Dec 9;8(12):e81609. doi: 10.1371/journal.pone.0081609 (PMC3857192; doi:10.1371/journal.pone.0081609)

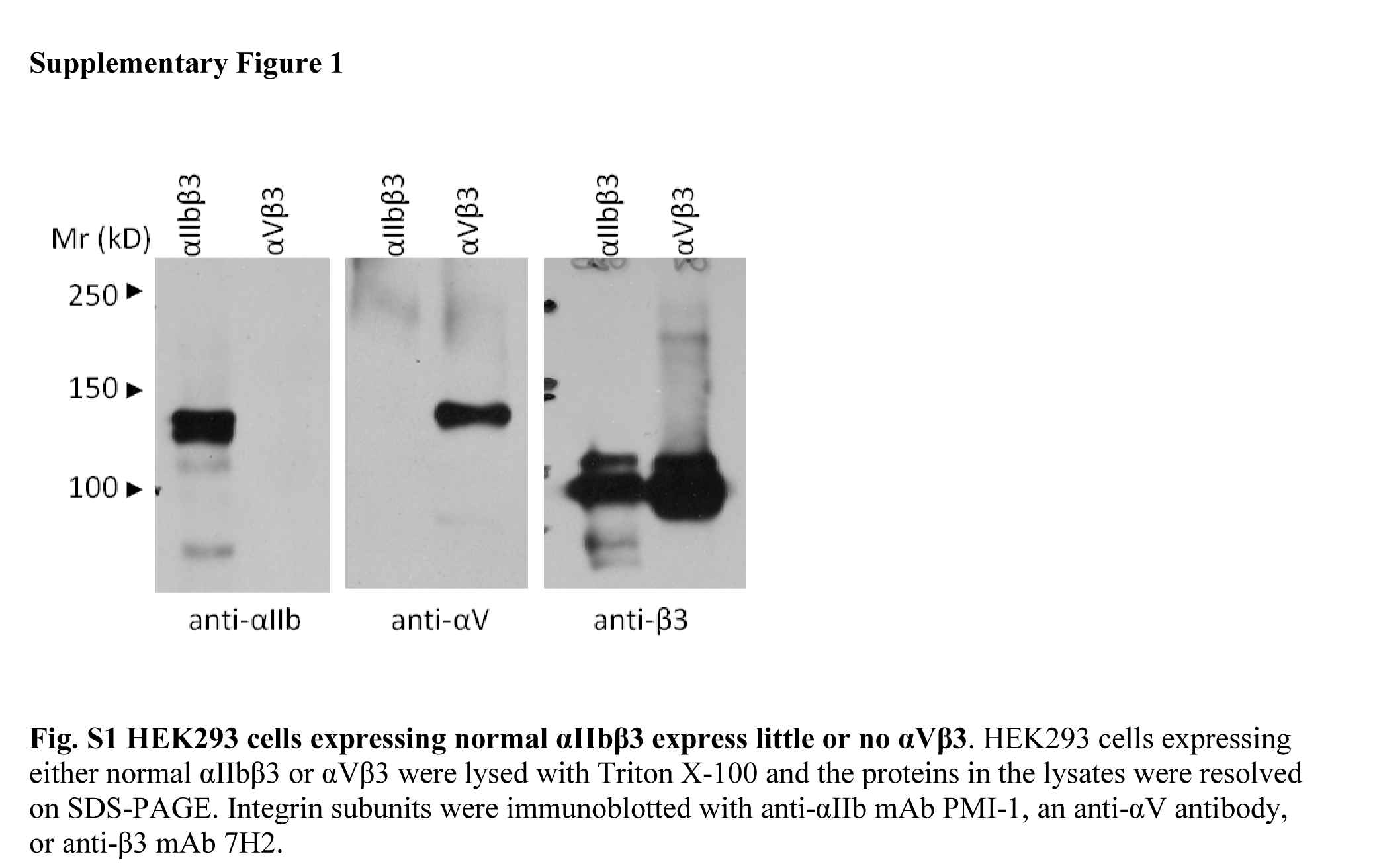

Supplement: Figure S1 — HEK293 cells expressing normal αIIbβ3 express little or no αVβ3. HEK293 cells expressing either normal αIIbβ3 or αVβ3 were lysed with Triton X-100 and the proteins in the lysates were resolved on SDS-PAGE. Integrin subunits were immunoblotted with anti-αIIb mAb PMI-1, an anti-αV antibody, or anti-β3 mAb 7H2. (TIF) [file pone.0081609.s001.tif]

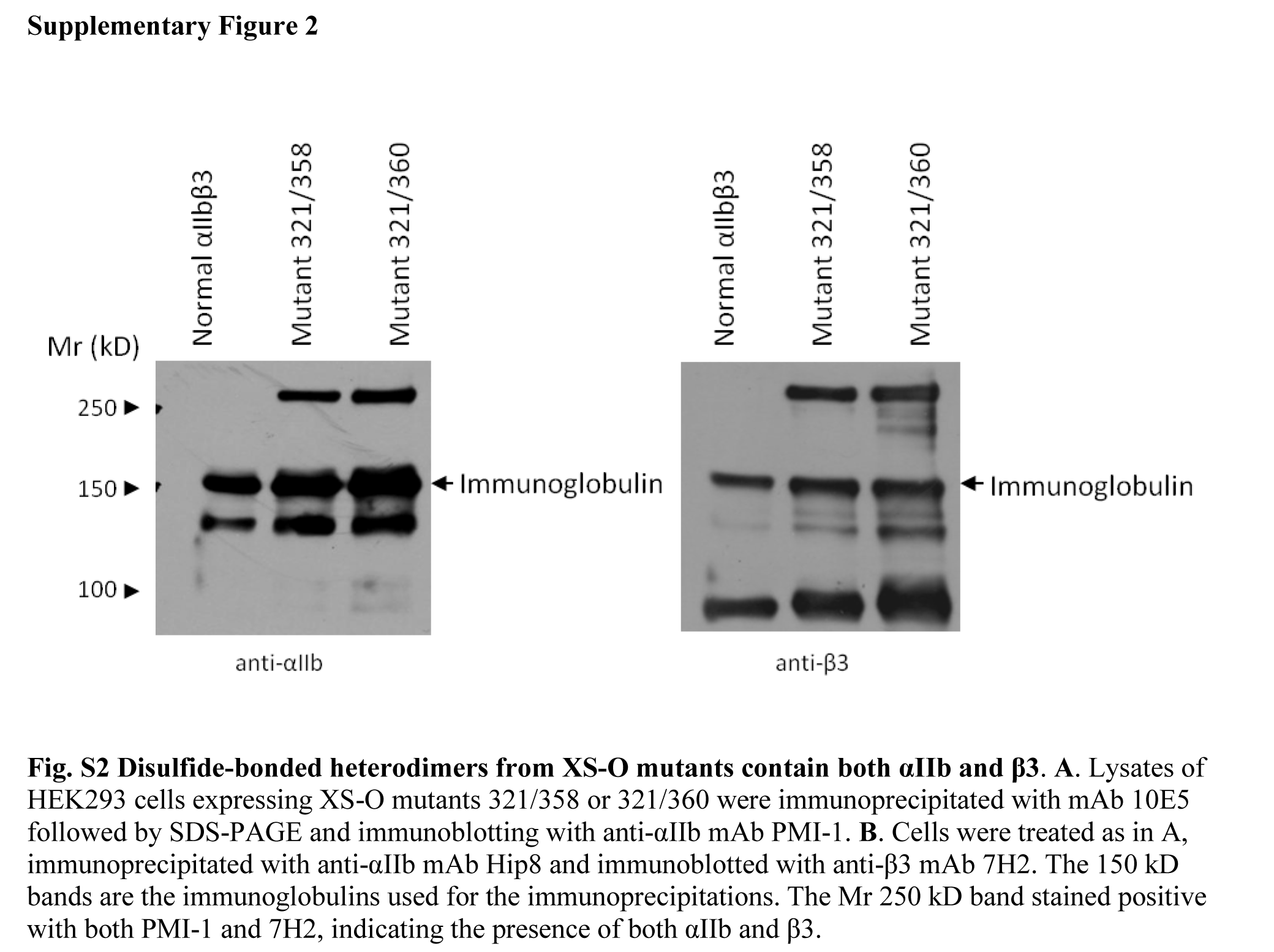

Supplement: Figure S2 — Disulfide-bonded heterodimers from XS-O mutants contain both αIIb and β3. A. Lysates of HEK293 cells expressing XS-O mutants 321/358 or 321/360 were immunoprecipitated with mAb 10E5 followed by SDS-PAGE and immunoblotting with anti-αIIb mAb PMI-1. B. Cells were treated as in A, immunoprecipitated with anti-αIIb mAb Hip8 and immunoblotted with anti-β3 mAb 7H2. The 150 kD bands are the immunoglobulins used for the immunoprecipitations. The Mr 250 kD band stained positive with both PMI-1 and 7H2, indicating the presence of both αIIb and β3. (TIF) [file pone.0081609.s002.tif]

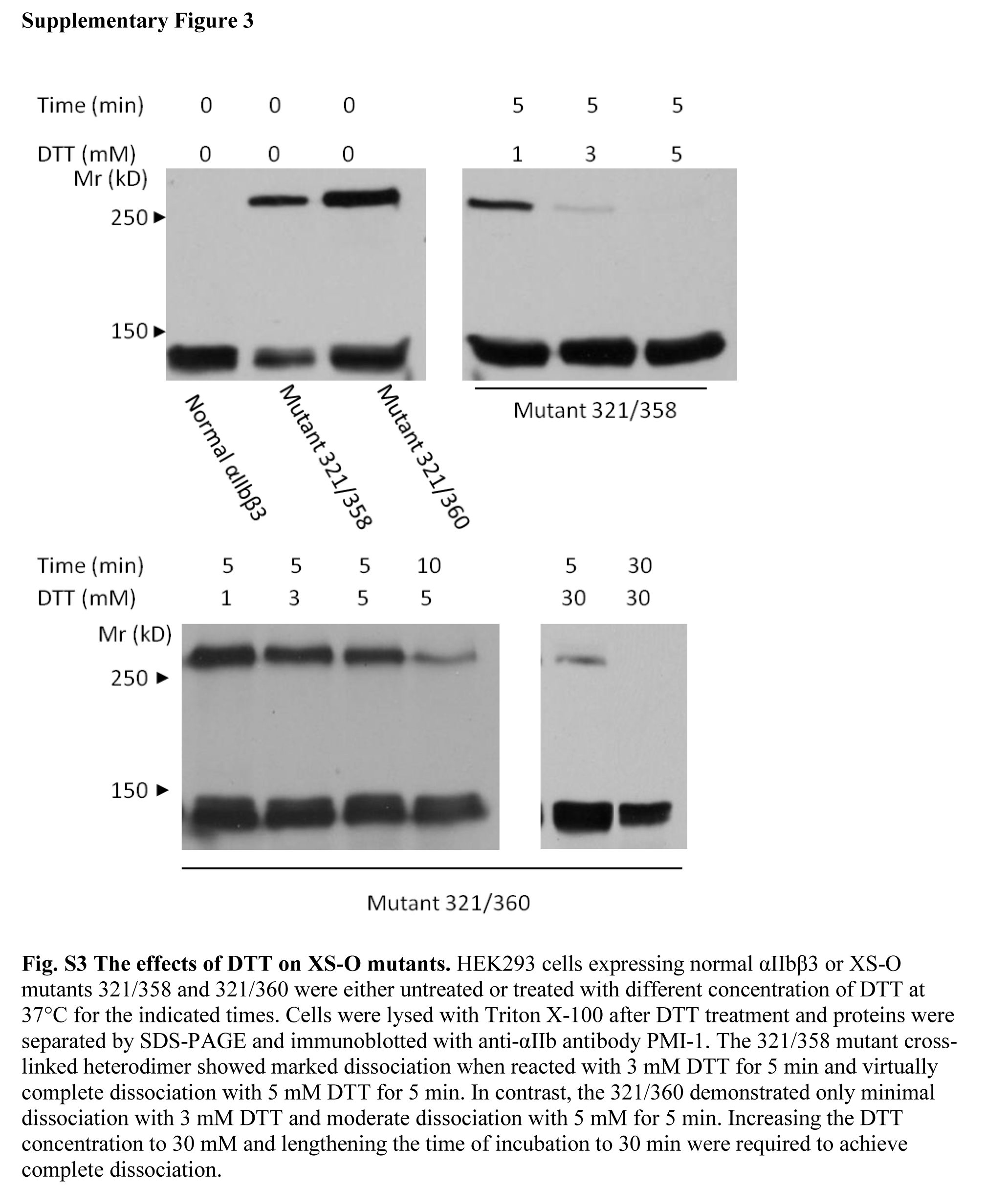

Supplement: Figure S3 — The effects of DTT on XS-O mutants. HEK293 cells expressing normal αIIbβ3 or XS-O mutants 321/358 and 321/360 were either untreated or treated with different concentration of DTT at 37°C for the indicated times. Cells were lysed with Triton X-100 after DTT treatment and proteins were separated by SDS-PAGE and immunoblotted with anti-αIIb antibody PMI-1. The 321/358 mutant cross-linked heterodimer showed marked dissociation when reacted with 3 mM DTT for 5 min and virtually complete dissociation with 5 mM DTT for 5 min. In contrast, the 321/360 demonstrated only minimal dissociation with 3 mM DTT and moderate dissociation with 5 mM for 5 min. Increasing the DTT concentration to 30 mM and lengthening the time of incubation to 30 min were required to achieve complete dissociation. (TIF) [file pone.0081609.s003.tif]

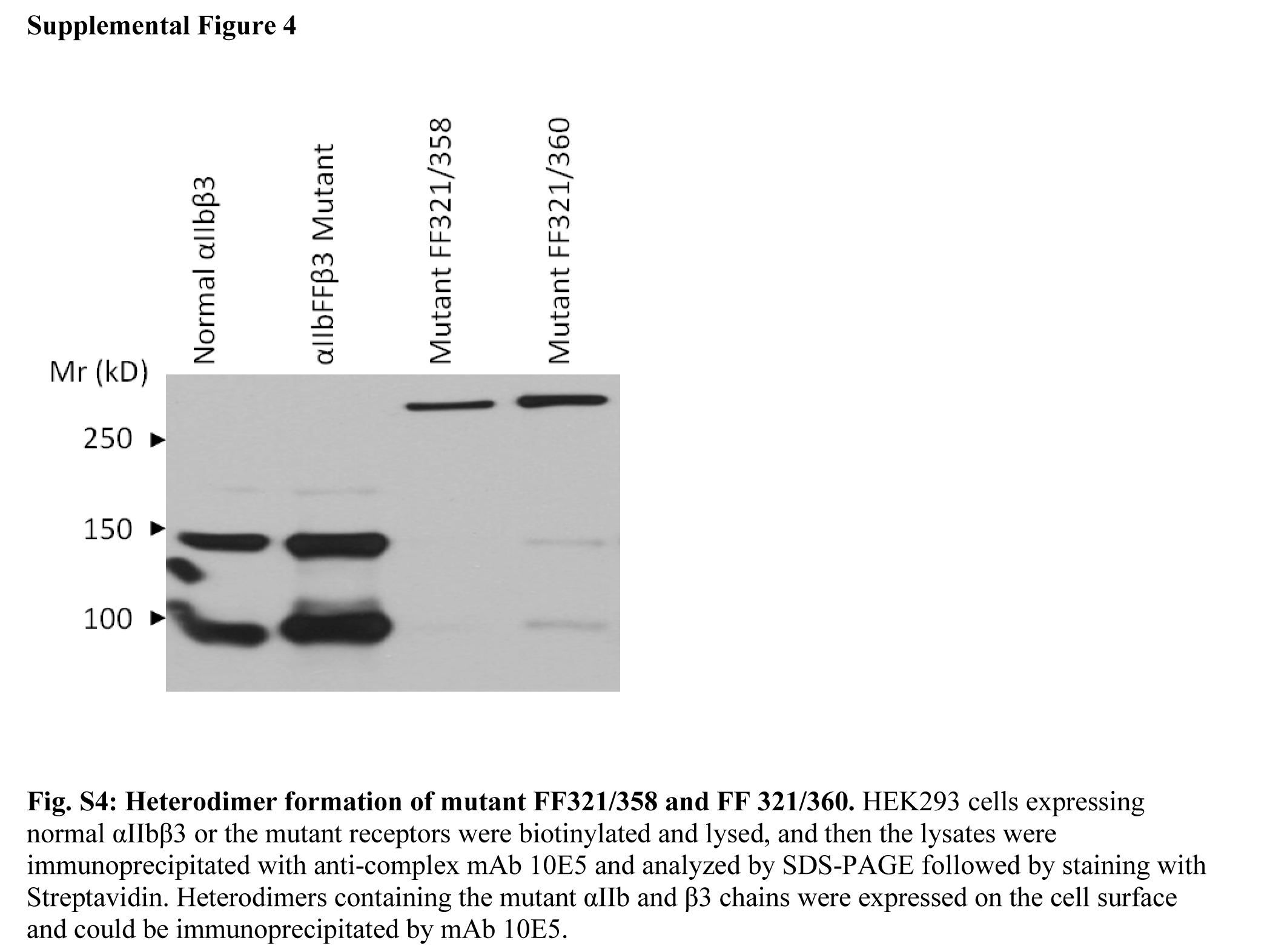

Supplement: Figure S4 — Heterodimer formation of mutant FF321/358 and FF 321/360. HEK293 cells expressing normal αIIbβ3 or the mutant receptors were biotinylated and lysed, and then the lysates were immunoprecipitated with anti-complex mAb 10E5 and analyzed by SDS-PAGE followed by staining with Streptavidin. Heterodimers containing the mutant αIIb and β3 chains were expressed on the cell surface and could be immunoprecipitated by mAb 10E5. (TIF) [file pone.0081609.s004.tif]

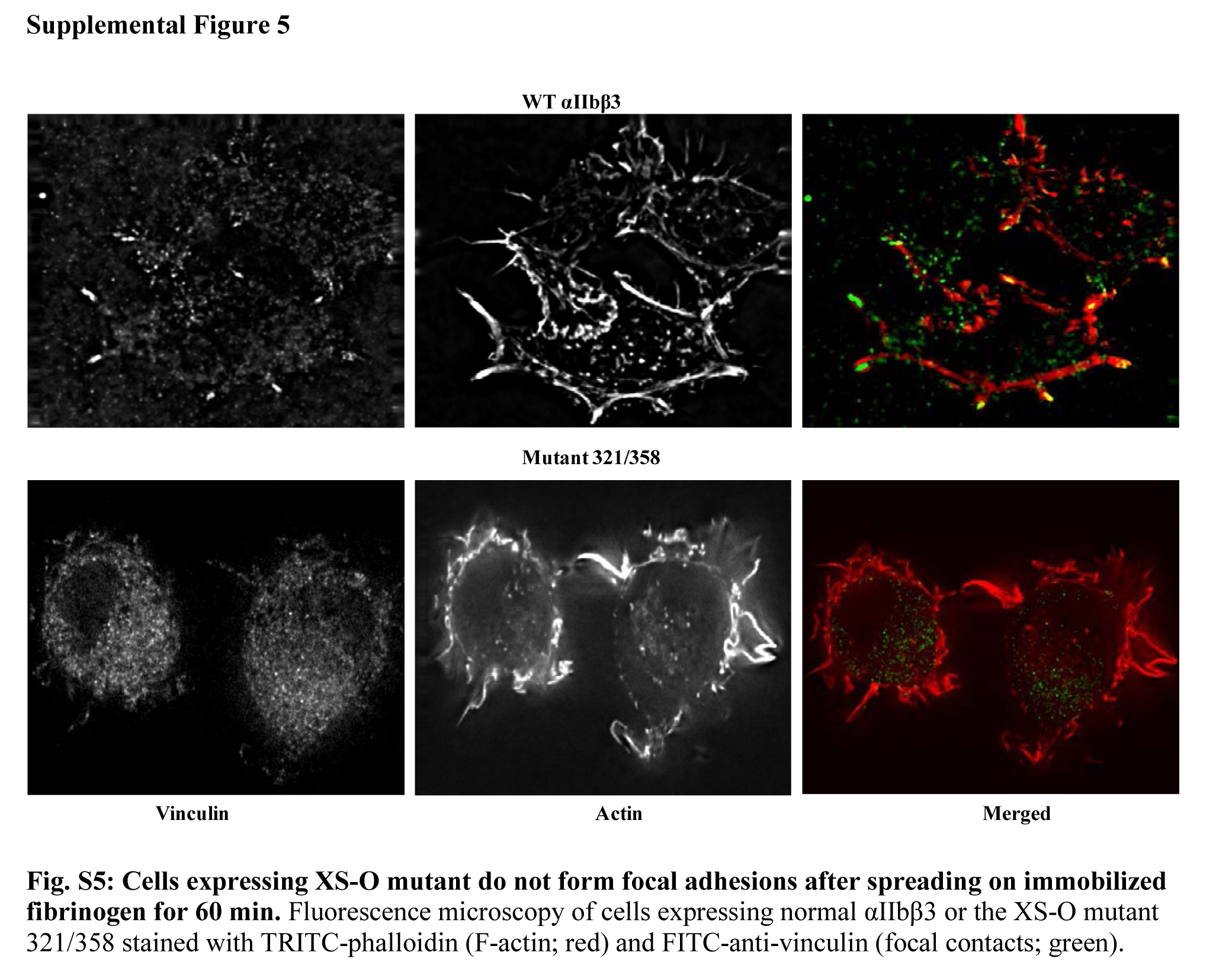

Supplement: Figure S5 — Cells expressing XS-O mutant do not form focal adhesions after spreading on immobilized fibrinogen for 60 min. Fluorescence microscopy of cells expressing normal αIIbβ3 or the XS-O mutant 321/358 stained with TRITC-phalloidin (F-actin; red) and FITC-anti-vinculin (focal contacts; green). (TIF) [file pone.0081609.s005.tif]

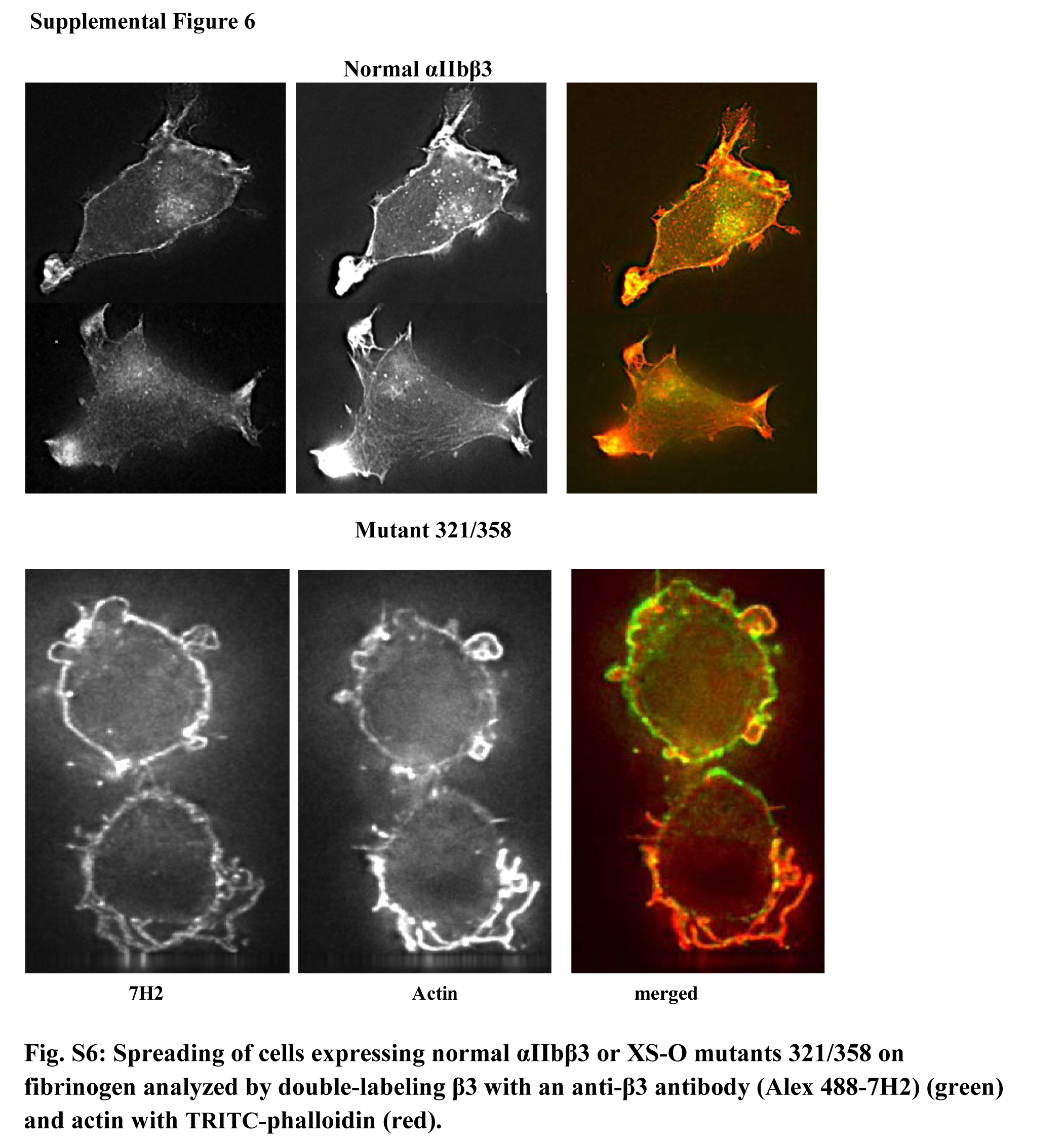

Supplement: Figure S6 — Spreading of cells expressing normal αIIbβ3 or XS-O mutants 321/358 on fibrinogen analyzed by double-labeling β3 with an anti-β3 antibody (Alex 488-7H2) (green) and actin with TRITC-phalloidin (red). (TIF) [file pone.0081609.s006.tif]
